# Supplementary material for: Multiscale hyperbolic embedding reveals hierarchical structure in complex biological systems
Source: NPJ Syst Biol Appl. 2026 Jun 2;12:130. doi: 10.1038/s41540-026-00725-z (PMC13530193; doi:10.1038/s41540-026-00725-z)
Supplement: Supplementary file 1 [file 41540_2026_725_MOESM1_ESM.pdf]

Supplementary materials

Pseudo code for MuH-MDS

Algorithm 1 shows the M-HMDS algorithm. In line 6, δ_{ij} is a pairwise distance matrix of samples in cluster m and cluster n; $MInd$ and $NInd$ indicates which samples belongs to cluster m or n. In line 13, $d_{i,\{n\}}$ is a pairwise distance matrix of sample i combined with samples in cluster n. In line 19, $\{\vec{y_k}\}_{NNs}$ is set of locations of cluster centroids of the nearest neighbors of cluster m. In line 25, $\{\vec{x_p}\}_{nns}$ is locations of nearest neighboring points of the outlier $\{\vec{x_p}\}$, and $d^{mutual}_{p,\{\vec{x_p}\}_{nns}}$ is mutual distance from outliers to them.

Algorithm 1 Multiscale hyperbolic multidimensional scaling (MuH-MDS)

Input Sample feature matrix $\{\vec{v_i}\}$ or pairwise distance matrix δ_{ij}, number of nearest neighbors α, number of hyperbolic space dimension D, maximal/minimal number of samples in each cluster $[n_{max}, n_{min}]$, Boolean variable $MapOutier$ determining whether to map outliers
Output Embedding coordinates in $\mathbb{H}^D$: $\{\vec{x_i}\}$
$ClusterIDs, OutlierIDs \leftarrow$ ClusteringAndFiltering($\{\vec{v_i}\}$ or d_{ij}, n_{max}, n_{min})
for m, n in Unique($ClusterIDs$) **do**
    $\delta_{mn} \leftarrow$ GetDmatFeature($\{\vec{v}\}_m$,$\{\vec{v}\}_n$)
    or GetDmatDistance($\delta_{ij}, MInd, NInd$)
end for
for m in Unique($ClusterIDs$) **do**
    $NNs \leftarrow \alpha$ nearest neighbors of cluster m
    for i in cluster m **do**
        for n in NNs **do**
            $\delta^{mutual}_{m,in} \leftarrow$ GetDmatFeature($\vec{v_i}, \{\vec{v}\}_n$)
            or GetDmatDistance($\delta_{i,\{n\}}, MInd = 0, NInd = [1, 2, ...len(d) - 1]$)
        end for
    end for
end for
$\{\vec{y_k}\} \leftarrow$ HMDS(δ_{mn})
for m in Unique($ClusterIDs$) **do**
    (In parallel) $\{\vec{x^m_i}\} \leftarrow$ Relax($\delta^{mutual}_{m,in}, \delta^{self}_{k,ij}, \{\vec{y_k}\}_{NNs}$)
end for
(comment: *HMDS* algorithm were described in the main text and BHMDS paper [1], *Relax* algorithm were described in Methods)
$\{\vec{x_i}\} \leftarrow \cup\{\vec{x^m_i}\}$
if $MapOutier$ **then**
    for p in Outliers **do**
        $\vec{x_p} \leftarrow$ Relax($d^{mutual}_{p,\{\vec{x_p}\}_{nns}}$,$\{\vec{x}_{p_{nns}}\}$)
        $\{\vec{x_i}\} \leftarrow \{\vec{x_i}\} \cup \{\vec{x_p}\}$
    end for
end if
return $\{\vec{x_i}\}$

Algorithm 2 simply defines the function for clustering, filtering and cluster size control.

Algorithm 2 ClusteringAndFiltering($\{\vec{v_i}\}$ or δ_{ij}, n_{max}, n_{min})

```
Input Sample feature matrix {v_i} or pairwise distance matrix δ_ij, maximal/minimal number of samples in each cluster [n_max, n_min]
Output Assignment of cluster for each sample ClusterIDs
ClusterIDs ← Cluster assignment of each sample, from clustering on feature matrix {v_i} or pairwise distance matrix δ_ij
ClusterIDs, OutlierIDs ← Remove clusters whose size is smaller than n_min, re-divide clusters whose size larger than n_max until their size is no greater than n_max
return ClusterIDs, OutlierIDs
```

Compute distance to clusters from distance matrix

Algorithm 3 and 4 defines two different functions to fill the needs for different input types: feature matrix or pair-wise distance matrix. In Algorithm 1, it will determine which distance computing function to implement depend on the input type.

Algorithm 3 GetDmatFeature($\{\vec{u}\}, \{\vec{v}\}$)

Input Two set of vectors from the same vector space $\{\vec{u}\}, \{\vec{v}\}$

Output d, Average distance between the two sets

$d \leftarrow$ |Mean($\{\vec{u}\}$)-Mean($\{\vec{v}\}$)|

return d

Algorithm 4 GetDmatDistance

Input Distance matrix D, two sets of unique index $I = \{i\}$ and $J = \{j\}$ with $I \cap J = \{\}$, and $|I \cup J|$=len(D). I and J indicate which points belongs to which of the two clusters.

Output d, Average distance between the two sets

for j in J **do**

  $d_{Ij} \leftarrow \text{sqrt}(\frac{1}{|I|}\sum_{i \in I} d_{ij} - \frac{1}{|I|^2}\sum_{i,i' \in I, i' < i} d_{i,i'})$

end for

$d \leftarrow \text{sqrt}(\frac{1}{|J|}\sum_{j \in J} d_{Ij} - \frac{1}{|J|^2}\sum_{j,j' \in J, j' < j} d_{j,j'})$

return d

The computation in Algorithm 4 is based on following approximations: Suppose we have a set of n vectors $\vec{y_i}$ and a vector $\vec{x}$. Let $\vec{d_i} = \vec{x} - \vec{y_i}$. The goal is to approximate $d = |\vec{x} - Mean(\vec{y_i})|$ from $d_i \equiv |\vec{x} - \vec{y_i}| = |\vec{d_i}|$ and $d_{ii'} \equiv |\vec{y_i} - \vec{y_{i'}}| = |\vec{d_i} - \vec{d_{i'}}|$.

We have

$$\begin{aligned} d &= |\vec{x} - \frac{1}{n}\sum_n \vec{y_i}| = \frac{1}{n}|\sum_n (\vec{x} - \vec{y_i})| = \frac{1}{n}|\sum_n (\vec{d_i})| \\ &= \frac{1}{n}\sqrt{\sum_i d_i^2 + 2\sum_{i<i'} \vec{d_i}\cdot\vec{d_{i'}}} \end{aligned}$$

Since $d_{ii'}^2 = |\vec{d_i} - \vec{d_{i'}}|^2 = d_i^2 + d_{i'}^2 - 2\vec{d_i}\cdot\vec{d_{i'}}$, we have $2\vec{d_i}\cdot\vec{d_{i'}} = d_i^2 + d_{i'}^2 - d_{ii'}^2$. Therefore:

$$\begin{aligned} d &= \frac{1}{n}\sqrt{\sum_i d_i^2 + \sum_{i<i'} d_i^2 + d_{i'}^2 - d_{ii'}^2} \\ &= \frac{1}{n}\sqrt{n\sum_i d_i^2 - \sum_{i<i'} d_{ii'}^2} \\ &= \sqrt{\frac{1}{n}\sum_i d_i^2 - \frac{1}{n^2}\sum_{i<i'} d_{ii'}^2} \end{aligned}$$

One might notice that Algorithm 4 depends on the order of performing summation on different clusters. We set the order by the variance of original local distance matrix, and sum over cluster with smaller variance first.

Approximation on distance between clusters that are not nearest neighbor clusters

The Möbius addition is:

$$\vec{u} \oplus \vec{v} = \frac{(1 + 2\vec{u}\cdot\vec{v} + |\vec{v}|^2)\vec{u} + (1 - |\vec{u}|^2)\vec{v}}{1 + 2\vec{u}\cdot\vec{v} + |\vec{u}|^2|\vec{v}|^2}$$

Therefore,

$$\begin{aligned} \vec{u_i^m} &= \vec{w_m} \oplus \vec{\epsilon_i^m} \\ &= \frac{(1 + 2\vec{w_m}\cdot\vec{\epsilon_i^m} + |\vec{\epsilon_i^m}|^2)\vec{w_m} + (1 - |\vec{w_m}|^2)\vec{\epsilon_i^m}}{1 + 2\vec{w_m}\cdot\vec{\epsilon_i^m} + |\vec{w_m}|^2|\vec{\epsilon_i^m}|^2} \\ &= \frac{(1 + 2\vec{w_m}\cdot\vec{\epsilon_i^m} + o(|\vec{\epsilon}|^2))\vec{w_m} + (1 - |\vec{w_m}|^2)\vec{\epsilon_i^m}}{1 + 2\vec{w_m}\cdot\vec{\epsilon_i^m} + o(|\vec{\epsilon}|^2)} \\ &\approx \frac{(1 + 2\vec{w_m}\cdot\vec{\epsilon_i^m})\vec{w_m} + (1 - |\vec{w_m}|^2)\vec{\epsilon_i^m}}{1 + 2\vec{w_m}\cdot\vec{\epsilon_i^m}} \\ &= \vec{w_m} + \frac{1 - |\vec{w_m}|^2}{1 + 2\vec{w_m}\cdot\vec{\epsilon_i^m}}\vec{\epsilon_i^m} \end{aligned}$$

Let $\alpha_i^m = \frac{1-|\vec{w_m}|^2}{1+2\vec{w_m}\cdot\vec{\epsilon_i^m}}$, then $\vec{u_i^m} = \vec{w_m} + \alpha_i^m \vec{\epsilon_i^m}$, upon dropping the $o(\epsilon^2)$ term.

Consider the scale of α_i^m: Since $\vec{w_m} \cdot \vec{\epsilon_i^m} \geq -|\vec{w_m}||\vec{\epsilon_i^m}|$, we have $\alpha_i^m = \frac{1-|\vec{w_m}|^2}{1+2\vec{w_m}\cdot\vec{\epsilon_i^m}} \leq \frac{1-|\vec{w_m}|^2}{1-2|\vec{w_m}||\vec{\epsilon_i^m}|}$. Given that $|\vec{\epsilon_i^m}| \ll |\vec{w_m}|$, so $2|\vec{\epsilon_i^m}| \ll |\vec{w_m}|$, we have $\alpha_i^m \leq \frac{1-|\vec{w_m}|^2}{1-2|\vec{w_m}||\vec{\epsilon_i^m}|} = \frac{1-|\vec{w_m}|^2}{1-|\vec{w_m}|\cdot 2|\vec{\epsilon_i^m}|} \ll \frac{1-|\vec{w_m}|^2}{1-|\vec{w_m}|\cdot|\vec{w_m}|} = 1$.

Since $\vec{u_i^m} = \vec{w_m} + \alpha_i^m \vec{\epsilon_i^m}$ and $\alpha_i^m \ll 1$, we have $\vec{u_i^m} = \vec{w_m} + o(|\vec{\epsilon_i^m}|)$.

The distance in Poincaré disk model is:

$$d^H(\vec{u}, \vec{v}) = \text{arccosh}(1 + 2\frac{|\vec{u} - \vec{v}|^2}{(1 - |\vec{u}|^2)(1 - |\vec{v}|^2)})$$

Therefore,

$$\begin{aligned}
d^H(\vec{u_i^m}, \vec{u_j^n}) &= d^H(\vec{w_m} + o(\epsilon), \vec{w_n} + o(\epsilon)) \\
&= \text{arccosh}(1 + 2\frac{|(\vec{w_m} + o(\epsilon)) - (\vec{w_n} + o(\epsilon))|^2}{(1 - |(\vec{w_m} + o(\epsilon))|^2)(1 - |(\vec{w_n} + o(\epsilon))|^2)}) \\
&= \text{arccosh}(1 + 2\frac{|\vec{w_m} - \vec{w_n}|^2 + o(\epsilon)}{(1 - |\vec{w_m}|^2 + o(\epsilon))(1 - |\vec{w_n}|^2 + o(\epsilon))}) \\
&\approx \text{arccosh}(1 + 2\frac{|\vec{w_m} - \vec{w_n}|^2}{(1 - |\vec{w_m}|^2)(1 - |\vec{w_n}|^2)}) \\
&= d^H(\vec{w_m}, \vec{w_n})
\end{aligned}$$

References

[1] Praturu, A. & Sharpee, T. O. Adaptive data embedding for curved spaces. *iScience* **27** (2024).

[2] Paul, F. *et al.* Transcriptional heterogeneity and lineage commitment in myeloid progenitors. *Cell* **163**, 1663–1677 (2015).

[3] De Sa, C., Gu, A., Ré, C. & Sala, F. Representation Tradeoffs for Hyperbolic Embeddings. *arXiv preprint arXiv:1804.03329* (2018).

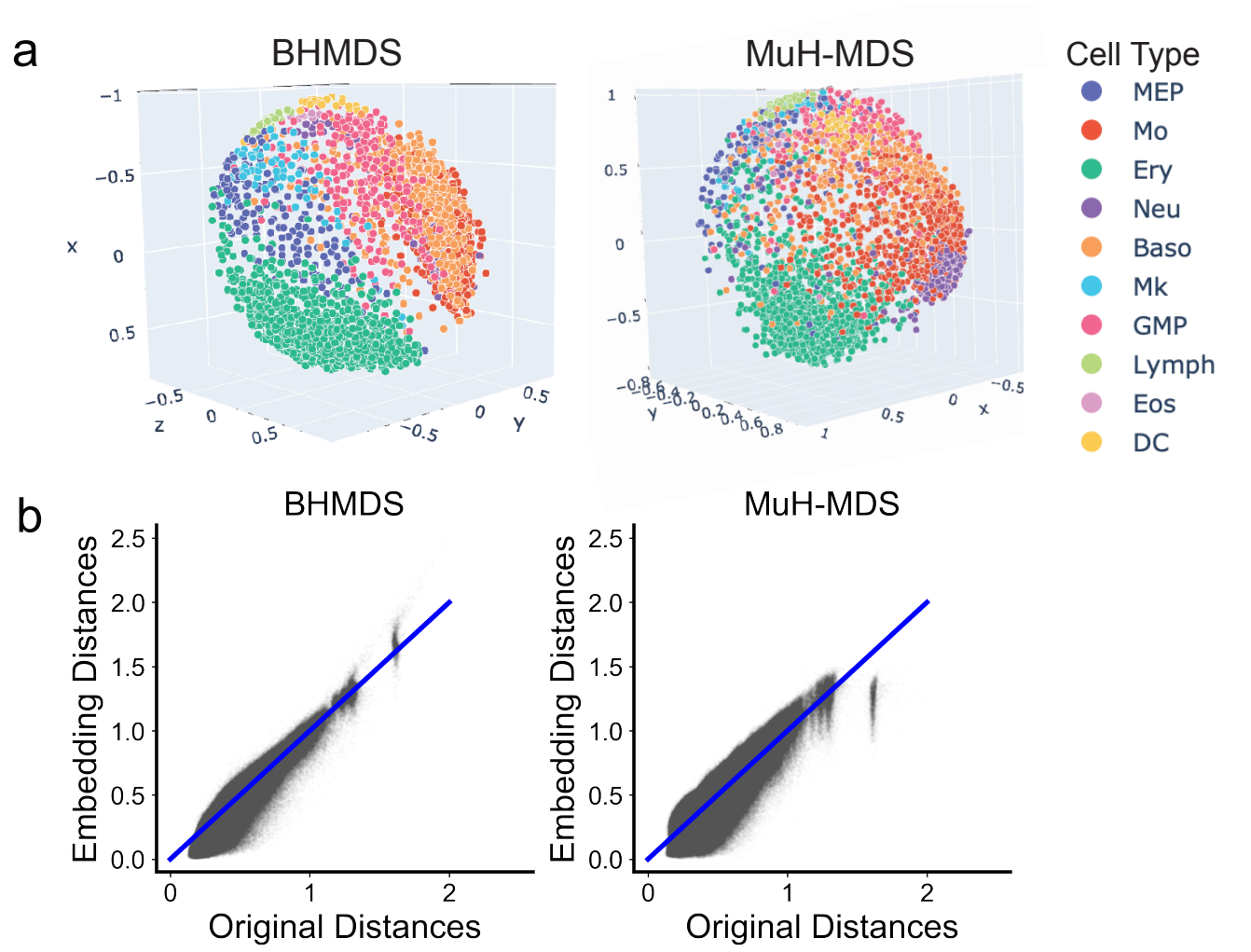

c

Metrics		BHMDS	MuH-MDS
Embedding Quality Metrics	Q-local	0.518583	0.414741
	Q-global	0.879617	0.826342
	Correlation	0.943675	0.855718
Hyperbolic Property	Curvature	7.947883	8.09052
Efficiency	Computing time (minutes)	**13806**	**15.00**

Figure S1: Comparison between the original (BHMDS) and multiscale (MuH-MDS) approaches using the mouse myeloid progenitors scRNA-seq dataset ("Paul") [2]. (a) Shepard diagram comparing original pairwise distances with embedded distances. (b) Embedding in 3-d hyperbolic space of "Paul" dataset. (c) Embedding metrics. MuH-MDS parameters: $\alpha = 30$, $k = 210$.

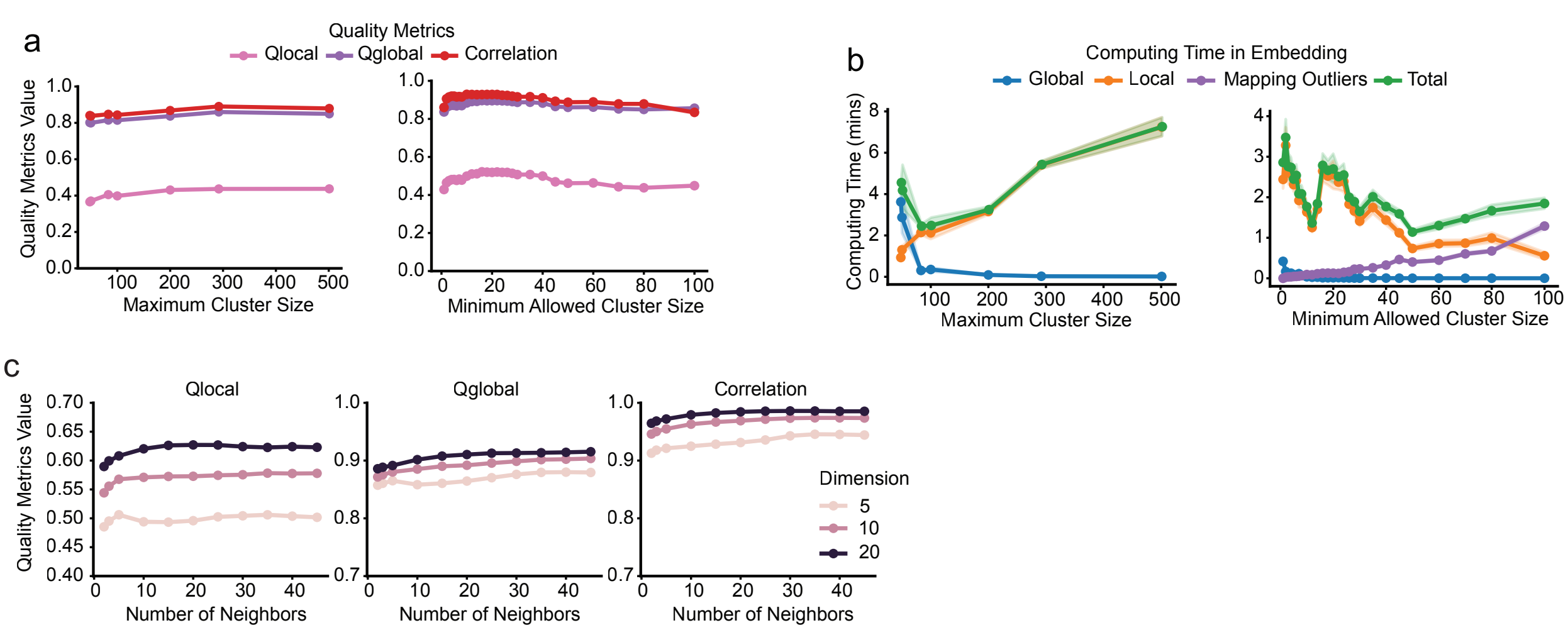

Figure S2: Simulation of hyperparameters using the Paul dataset [2], with 20 repetitions for each parameter setting. (a-b) Changes in embedding quality metrics (a) and running time (b) with varying maximum (left) or minimum (right) population sizes. (c) Embedding quality metrics across different numbers of nearest neighbors (α) in dimensions 5, 10, and 20. MuH-MDS parameters: (1) For maximum cluster size simulations: $k = 25$ $\alpha = 20$ $D = 3$; (2) For minimum allowed cluster size simulations: $k = 100$ $\alpha = 20$ $D = 3$; (3) For α and D simulations: $k = 50$.

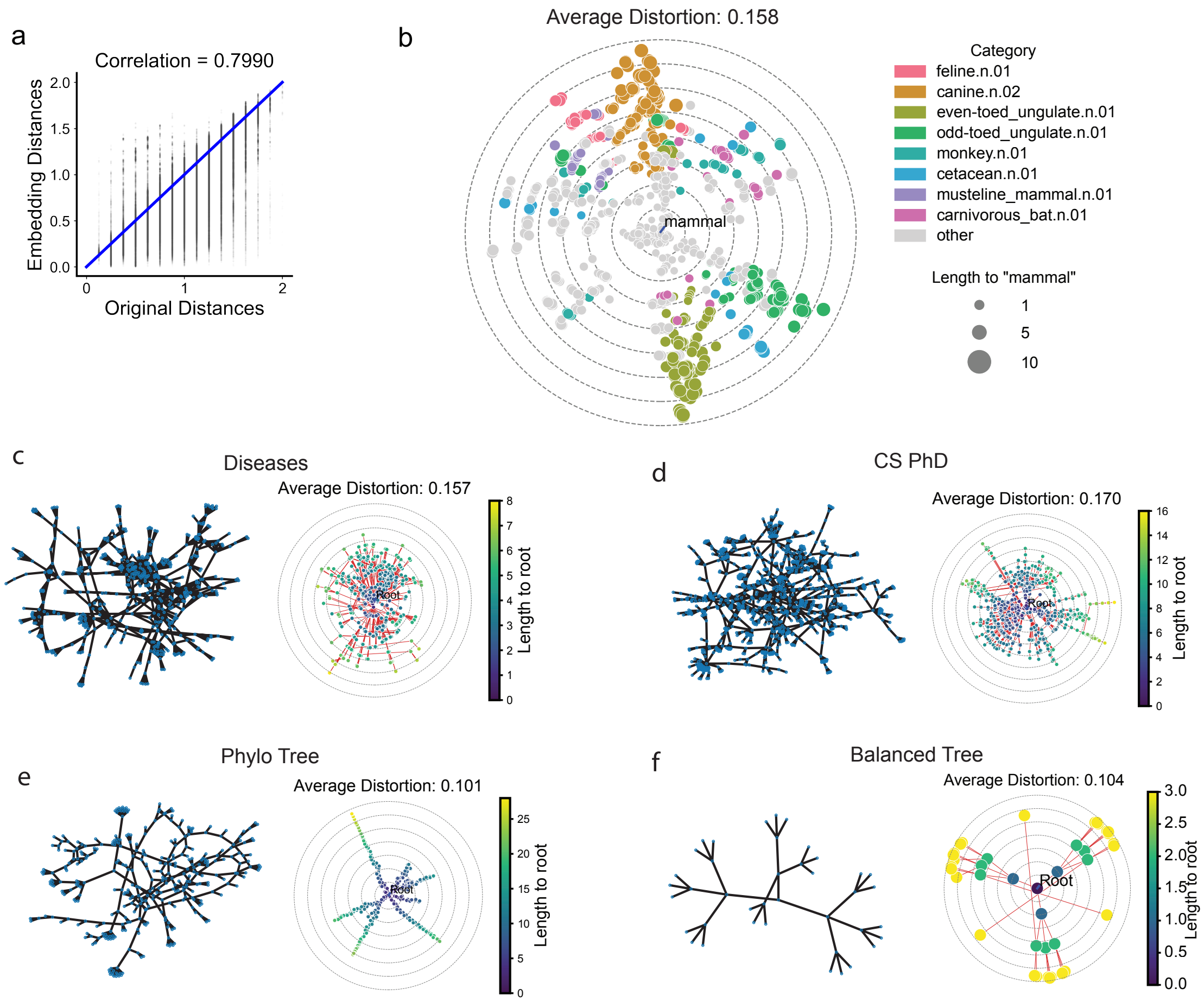

Figure S3: (a) Shepard diagram of WordNet mammal subtree embedding in 2-d hyperbolic space. (b) Application of MuH-MDS to the WordNet mammal subtree, shown in native coordinates. Marker size reflects the graph distance to the root node "mammal", while color denotes membership in semantic categories as shown in the legend. These categories correspond to parent nodes with the largest numbers of child nodes and were selected to highlight major structural branches within the hierarchy. MuH-MDS parameters: $k = 198$, $\alpha = 60$. (c-f) Embedding of multiple graph datasets from De Sa et al.[3] using MuH-MDS into 2D hyperbolic space. Left: original graph representations. Right: embedding results in 2D hyperbolic space. Color indicates graph distance to the root. Root is defined as the node with the smallest average distance to all other nodes in the original graph. MuH-MDS parameters: Diseases: $k = 106, \alpha = 60$, CS PhD: $k = 75, \alpha = 60$, Phylo Tree: $k = 34, \alpha = 20$, Balanced Tree: $k = 40, \alpha = 20$. For comparison, the distortion rates obtained by HoroPCA are: Diseases: 0.15, CS PhD: 0.16, Phylo Tree: 0.13, Balanced Tree: 0.19. MuH-MDS obtained comparable or better results on each dataset.

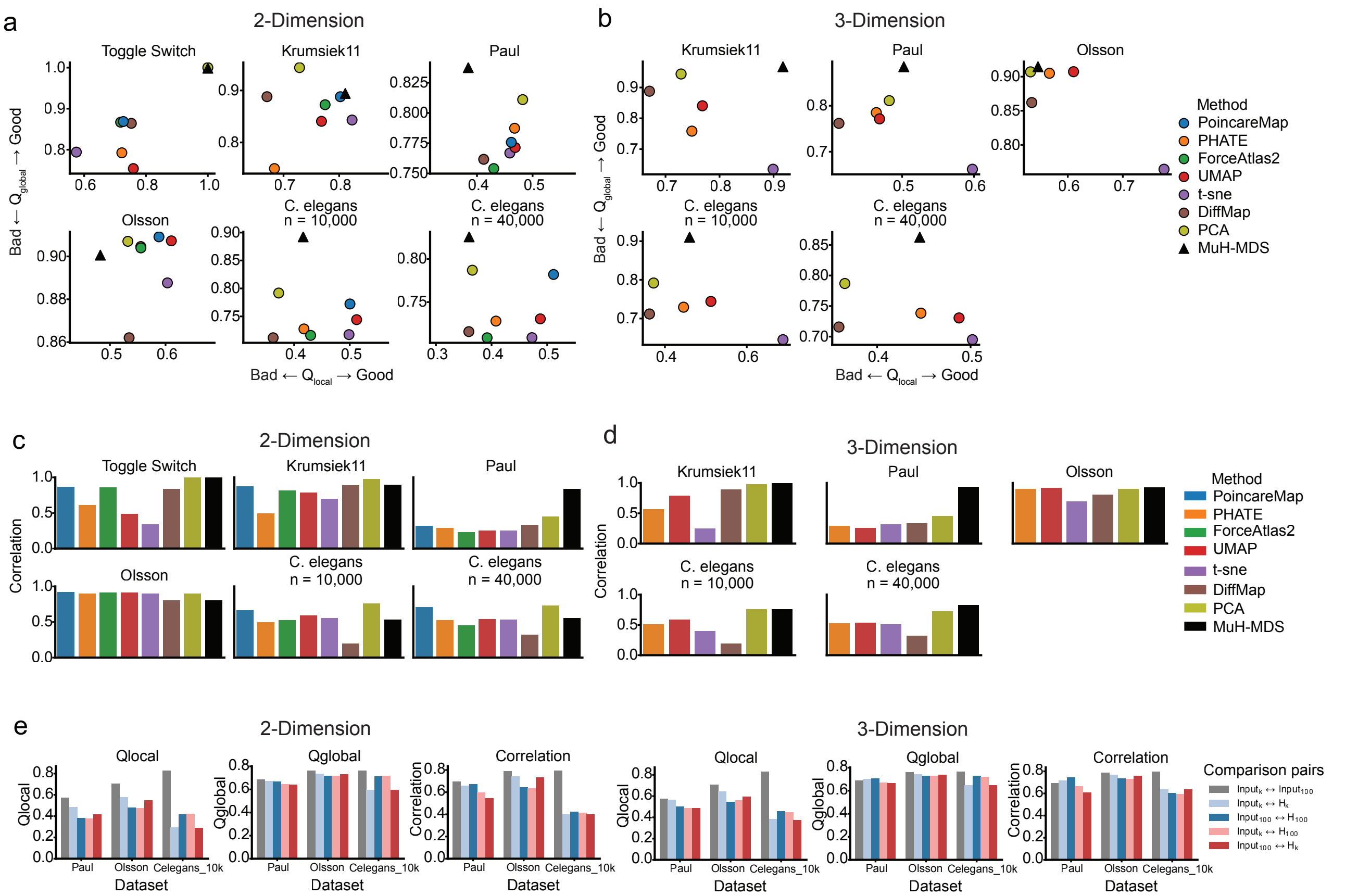

Figure S4: (a-d) Embedding quality metric for all datasets tested in the paper, in 2-dimension (a-b) or 3-dimension (c-d) space. MuH-MDS parameters: Toggle switch, 2d: $\alpha = 10, k = 20$. Krumsiek11, 2d: $\alpha = 10, k = 50$; 3d: $\alpha = 20, k = 50$. Paul, 2d and 3d: $\alpha = 20, k = 100$. Olsson, 2d: $\alpha = 30, k = 40$; 3d: $\alpha = 20, k = 40$. *C. elegans* (10,000) , 2d: $\alpha = 20, k = 300$; 3d: $\alpha = 30, k = 300$. *C. elegans* (40,000) , 2d and 3d: $\alpha = 30, k = 200$. (α: number of neighbors, k: number of clusters) (e) Robustness of representation structure to PCA dimensionality in preprocessing. Bars show pairwise embedding quality scores computed between different representation pairs. Input_k and Input_{100} denote PCA-reduced Euclidean input spaces retaining k and 100 principal components, respectively ($k = 20$ for Paul and Olsson, and $k = 60$ for *C. elegans*). H_k and H_{100} denote hyperbolic embeddings learned from Input_k and Input_{100}. Matched ($\mathrm{Input}_k \leftrightarrow \mathrm{H}_k$, $\mathrm{Input}_{100} \leftrightarrow \mathrm{H}_{100}$) and cross ($\mathrm{Input}_k \leftrightarrow \mathrm{H}_{100}$, $\mathrm{Input}_{100} \leftrightarrow \mathrm{H}_k$) comparisons assess the sensitivity of the learned representations to the number of retained PCs. Similar values observed for matched and cross comparisons indicate that the learned hyperbolic representations are robust to the choice of PCA dimensionality.

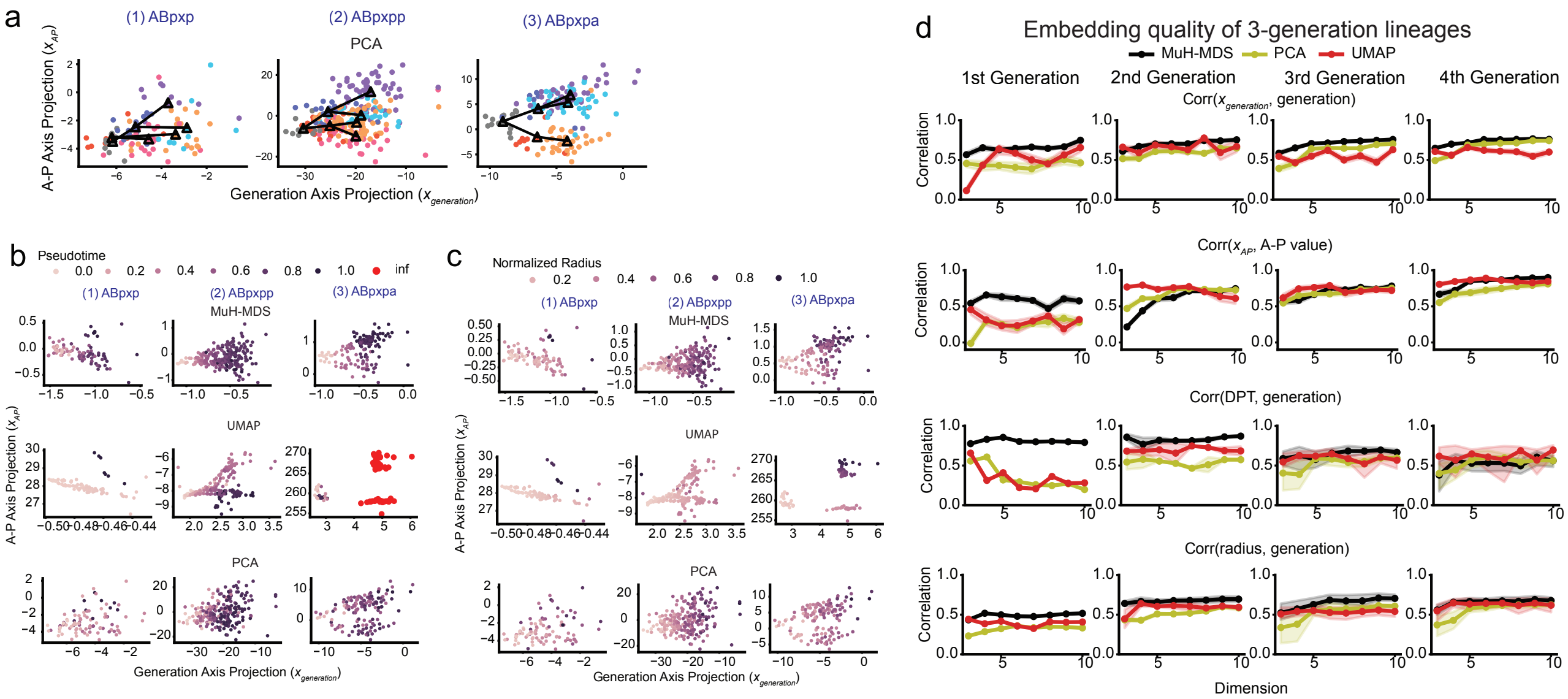

Figure S5: Analysis of the ABpxp lineage (7,562 samples) of *C. elegans* dataset. (a) 7-d PCA projecting to 2-d space, for 3-generation lineages starting from ABpxp, ABpxpp and ABpxpa. (b) Pseudotime computed from 7-d embedding of MuH-MDS, UMAP and PCA, for 3-generation lineages starting from ABpxp, ABpxpp and ABpxpa respectively. Some samples have infinite pseudotime because graph could be disconnected in high dimensional UMAP. (c) Normalize, re-centered radius computed from same embeddings as in (b). All embeddings are re-centered so that its population centroid is at the origin before computing the radius. (d) Comparison of 4 sub-lineage embedding quality metrics: (1) correlation between generation and projected coordinates (Corr($x_{\text{generation}}$, generation)), (2) correlation between A-P value and projected coordinates (Corr(x_{AP}, A-P value)), (3) correlation between generation and diffusion pseudotime inferred from embedding (Corr(DPT, generation)) and (4) correlation between generation and sample radius in embeddings (Corr(radius, generation)), across different dimensions, embedding methods (MuH-MDS, PCA, and UMAP), for lineages starting from 1st generation to 4th generation.

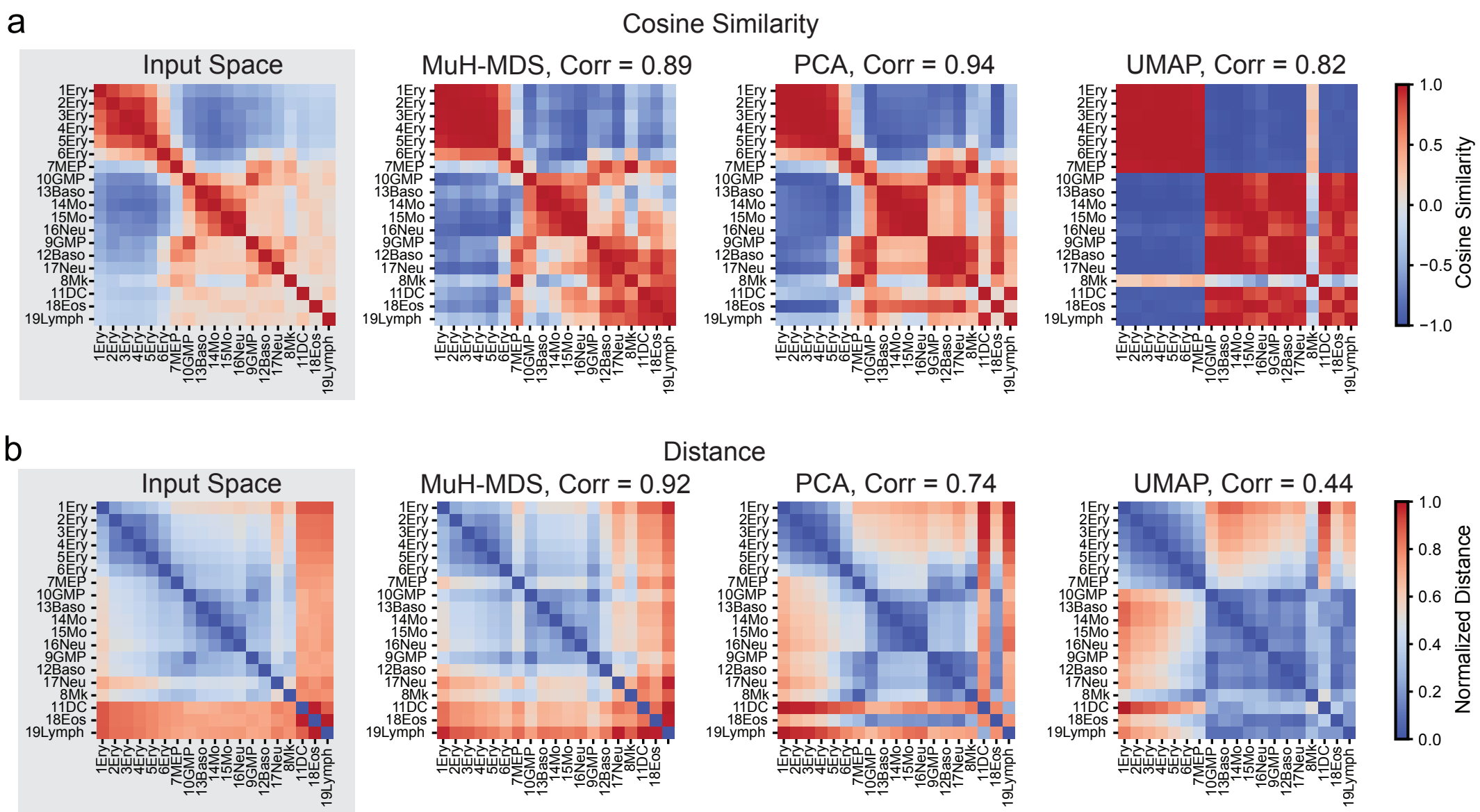

Figure S6: (a-b) Geometric comparison between the input space (100-PC PCA space derived from the original RNA-seq data) and each three-dimensional embedding, for all 19 clusters in "Paul" dataset. MuH-MDS best preserves both (a) cosine similarity and (b) pairwise distances from the input space.

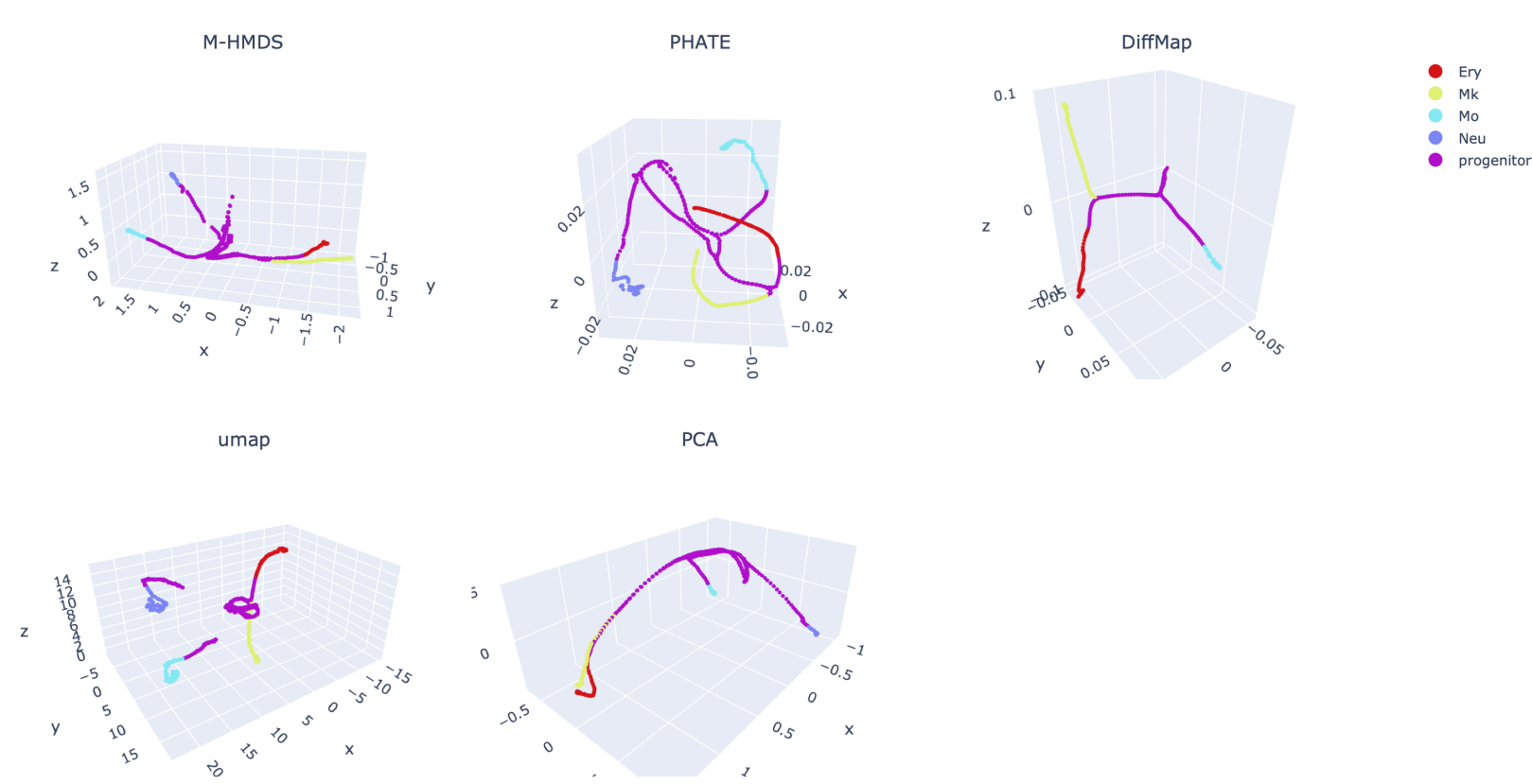

Figure S7: Embedding of the (synthetic) myeloid progenitors dataset (Krumsiek11), in 3-d space.

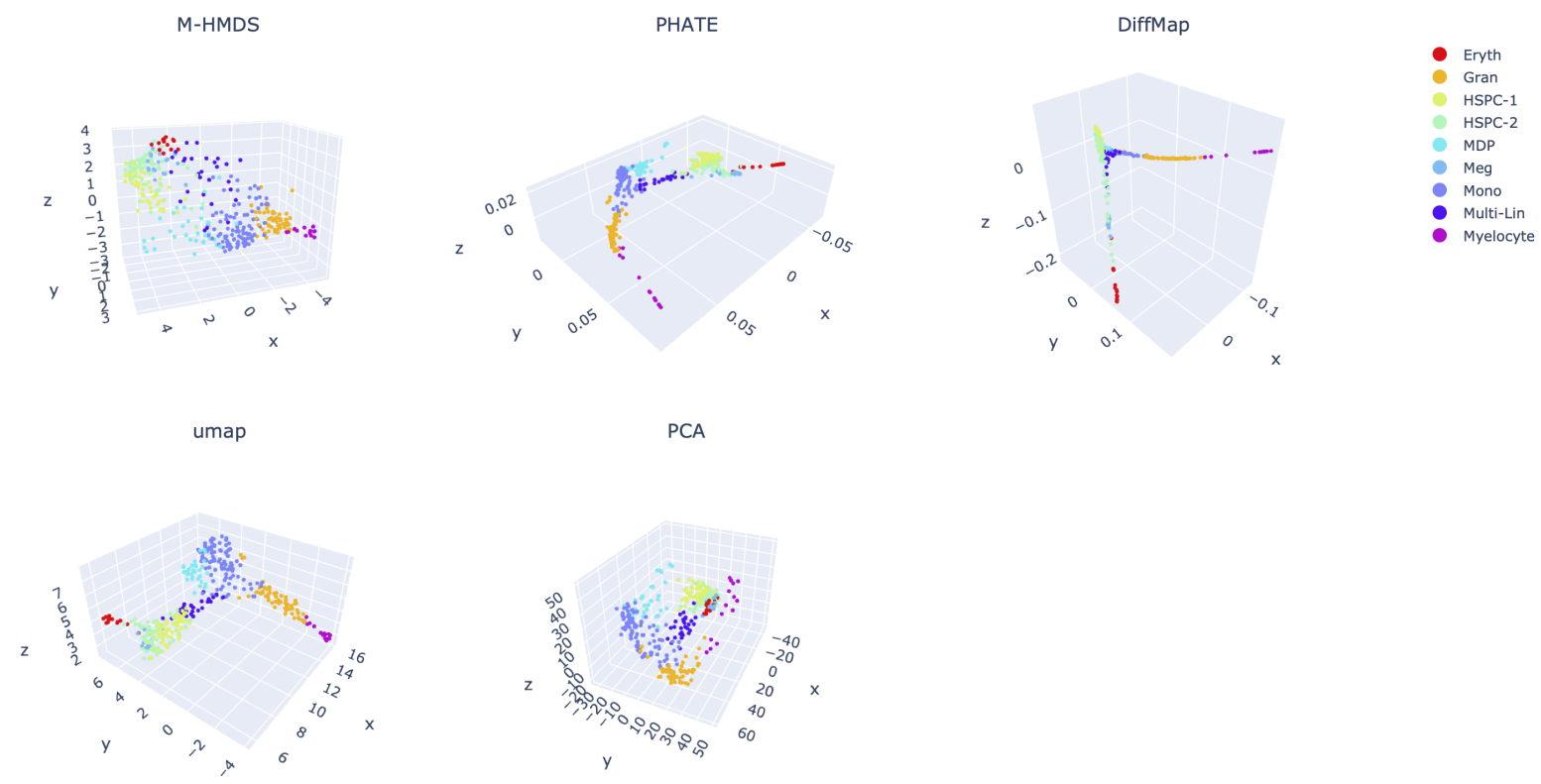

Figure S8: Embedding of the mouse myelopoesis dataset (Olsson), in 3-d space.

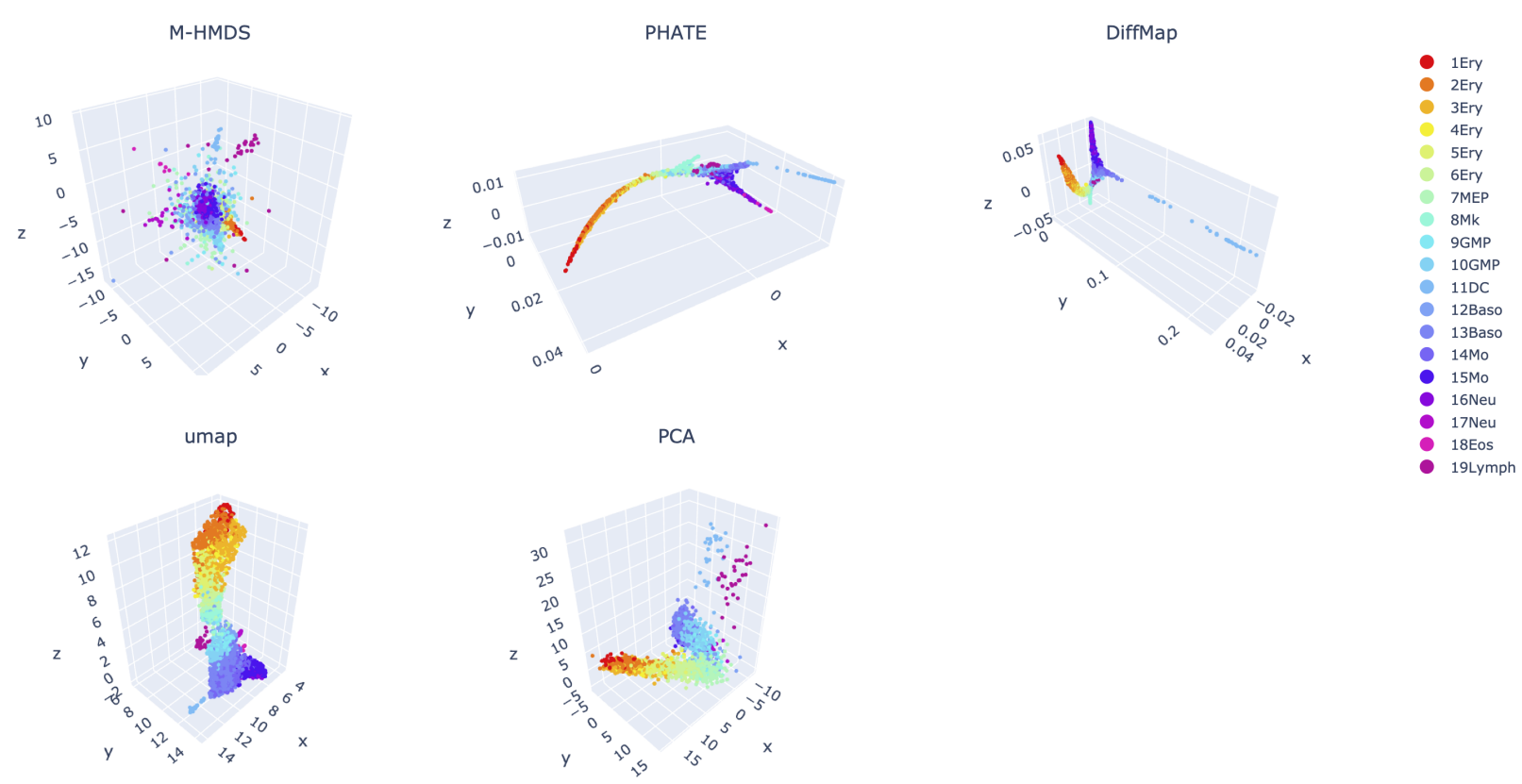

Figure S9: Embedding of the mouse myeloid progenitors dataset (Paul), in 3-d space.

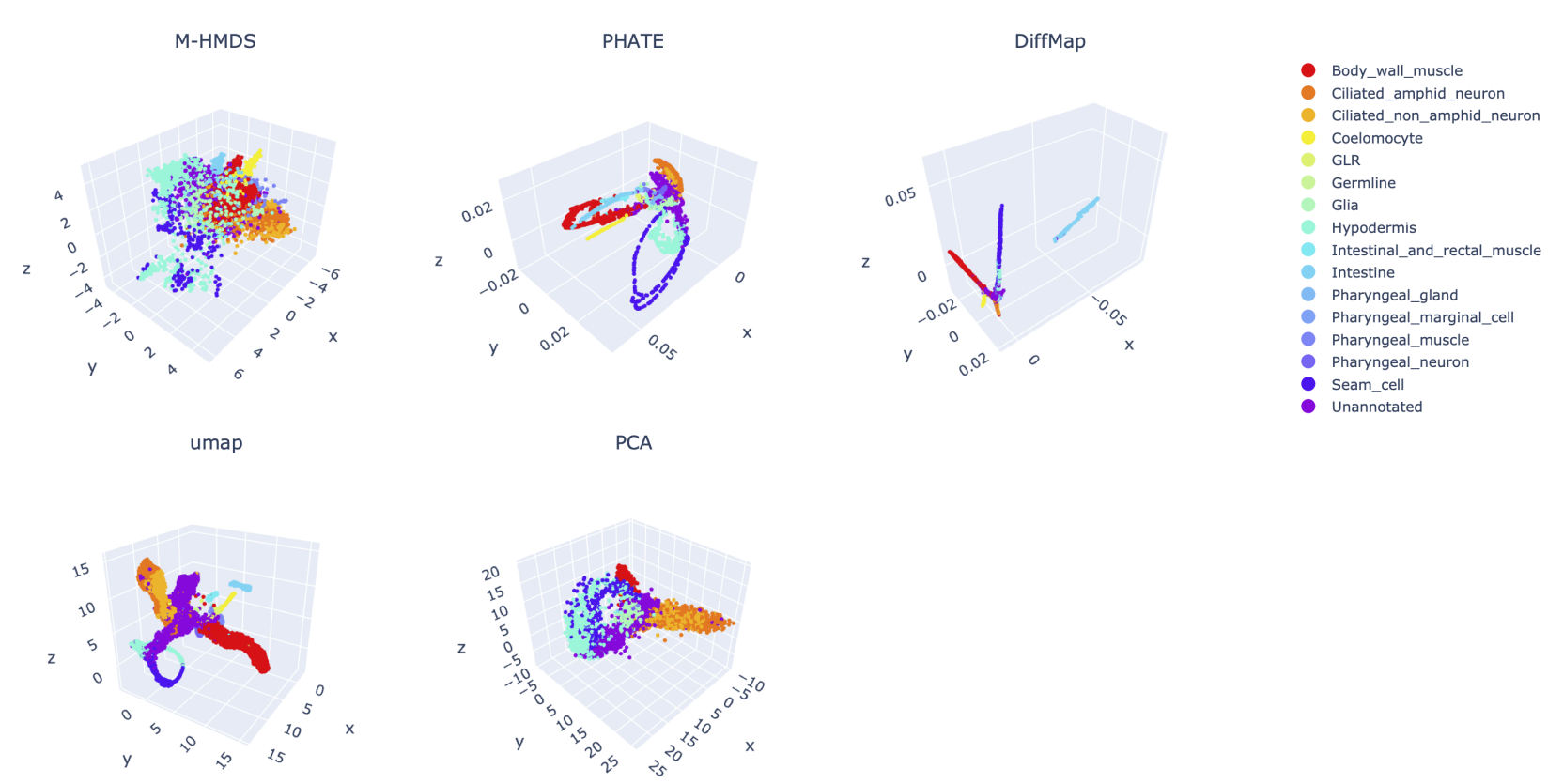

Figure S10: Embedding of the 10,000 sample subset of *C. elegans* dataset, in 3-d space, colored by cell types.

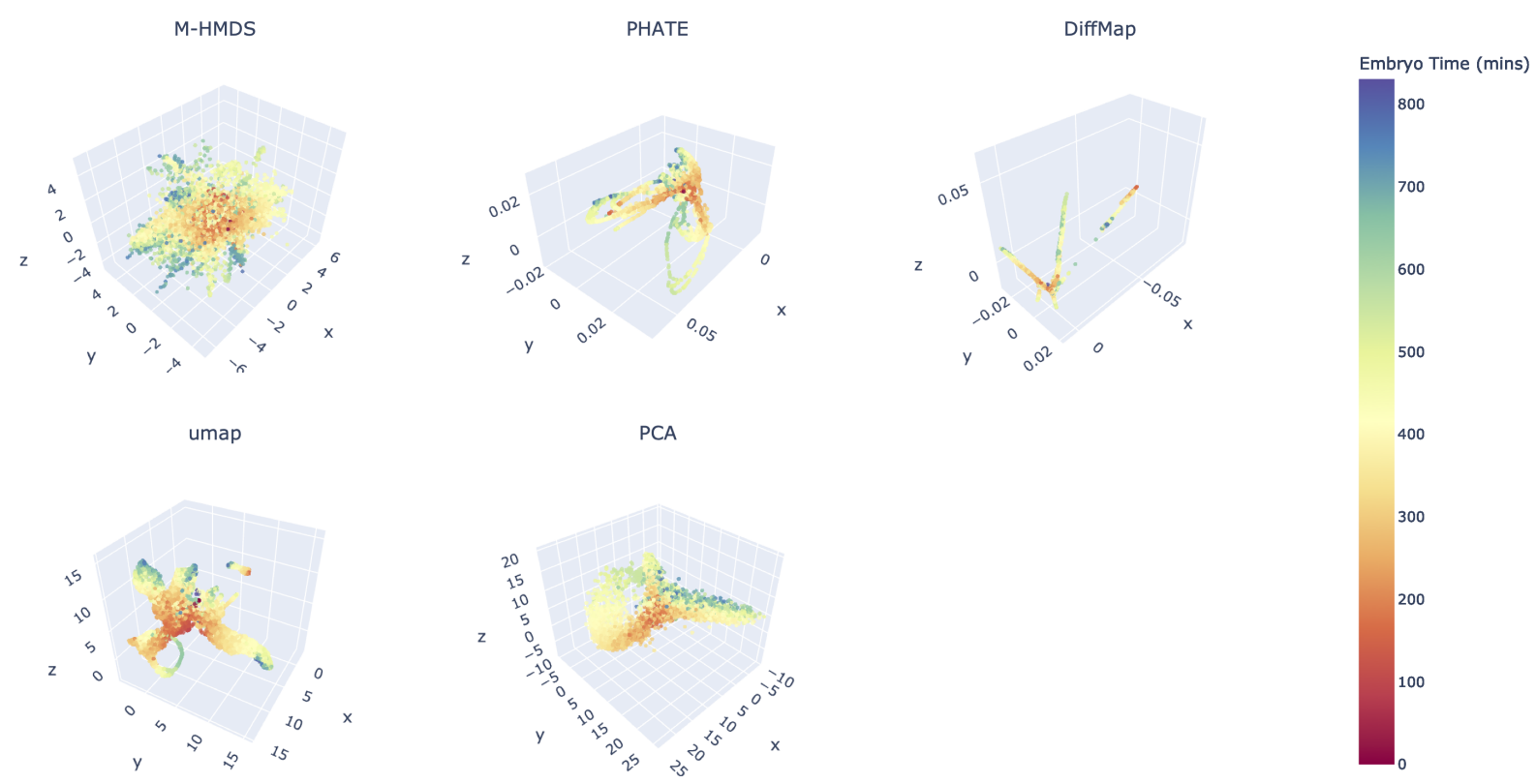

Figure S11: Same as Fig. (S10), but colored by embryo time.

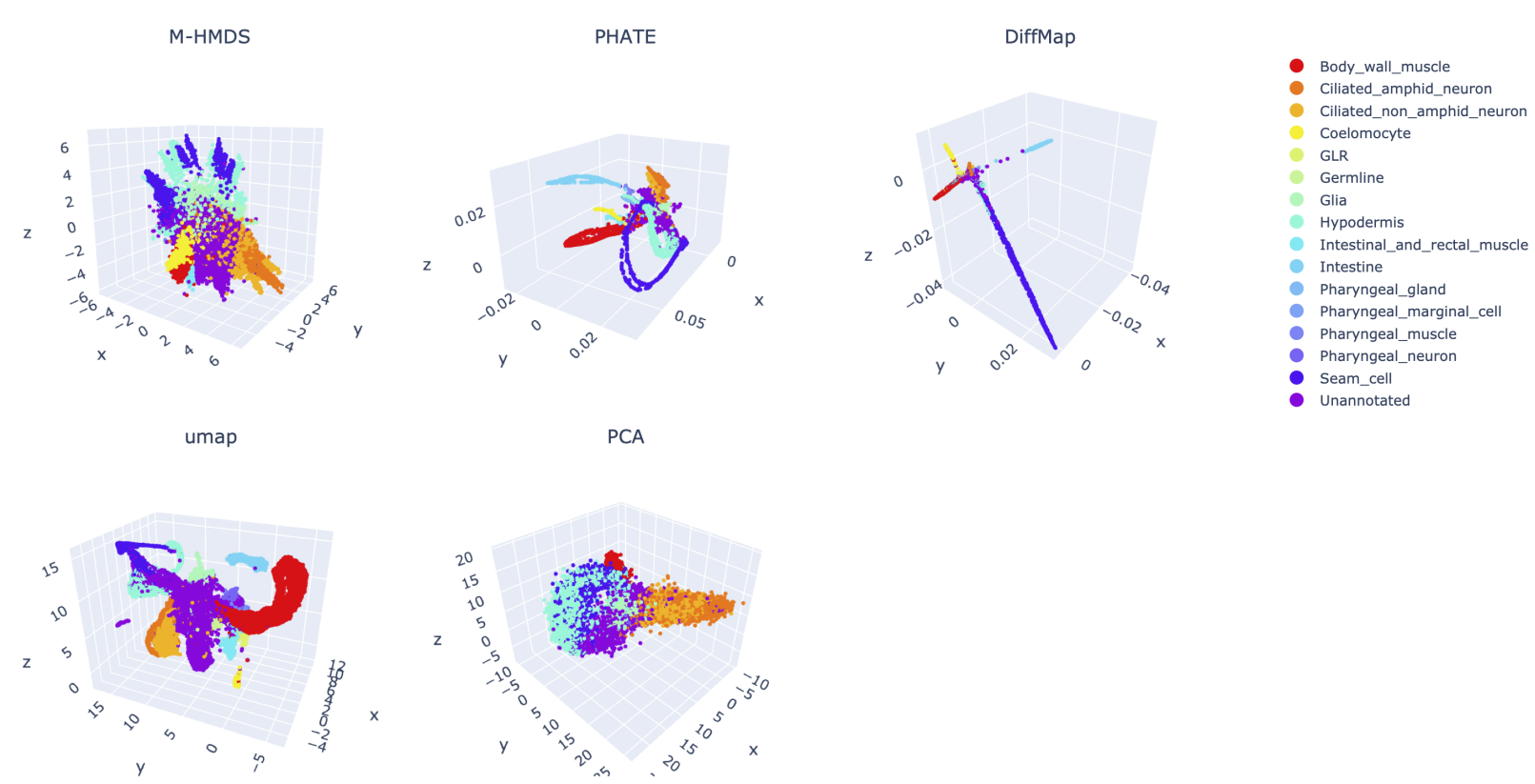

Figure S12: Embedding of the 40,000 sample subset of *C. elegans* dataset, in 3-d space, colored by cell types.

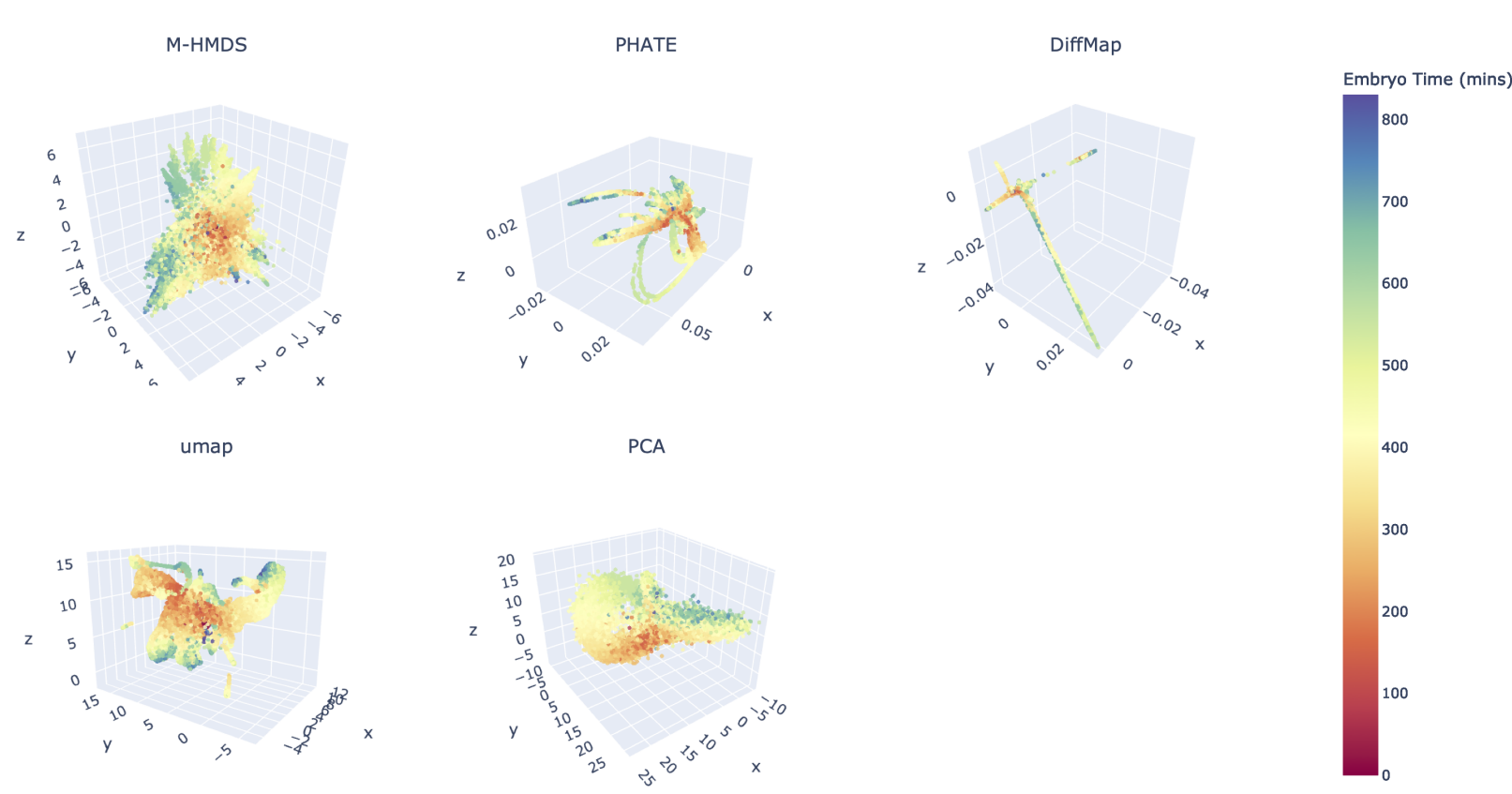

Figure S13: Same as Fig. (S12), but colored by embryo time.

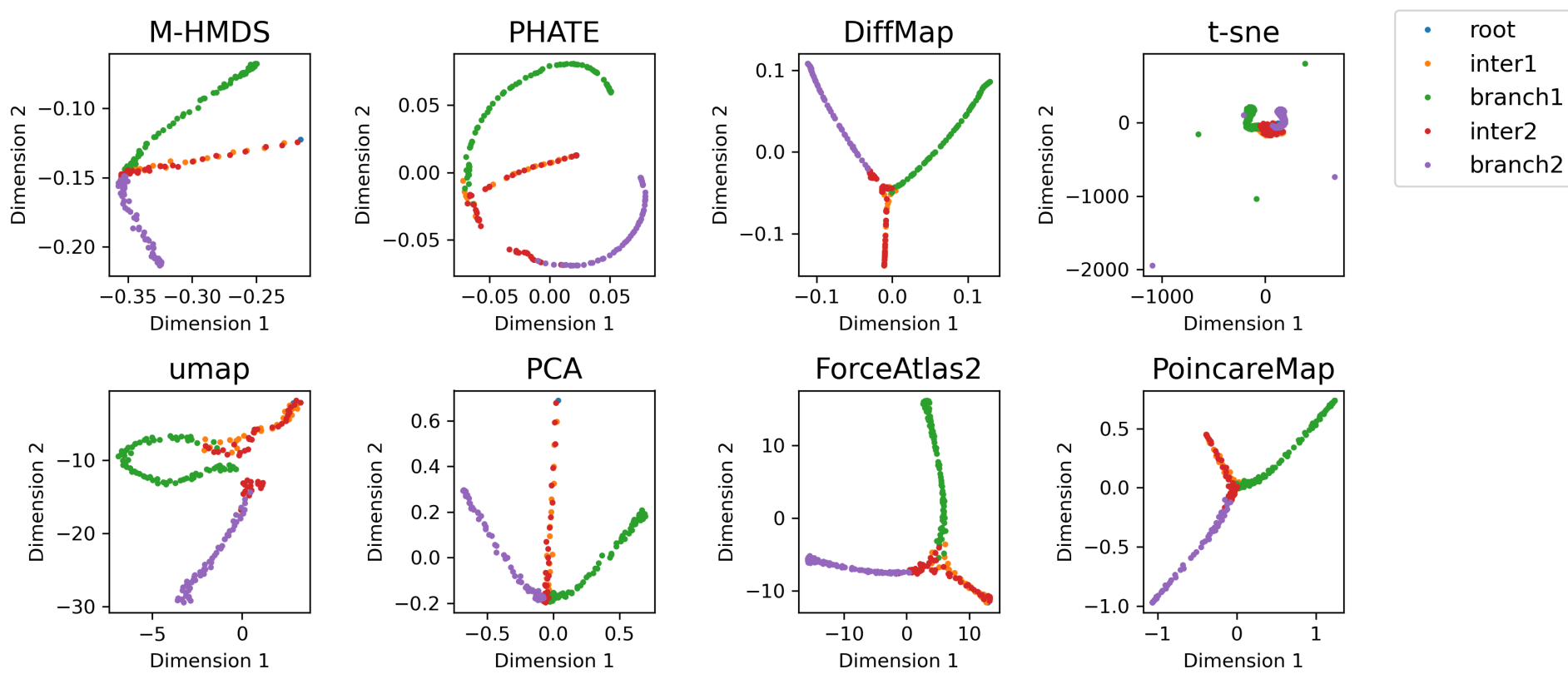

Figure S14: Embedding of the (synthetic) toggle switch dataset, in 2-d space.

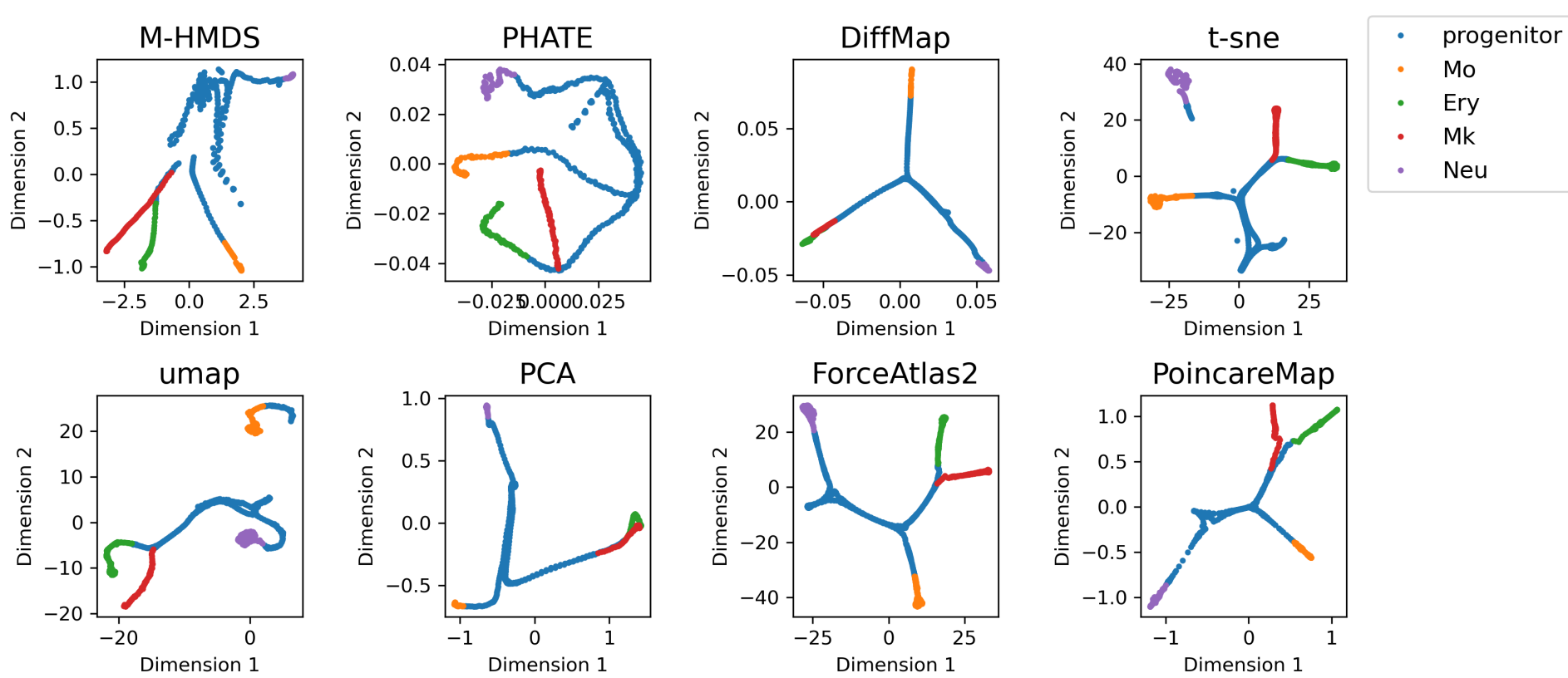

Figure S15: Embedding of the (synthetic) myeloid progenitors dataset (Krumsiek11), in 2-d space.

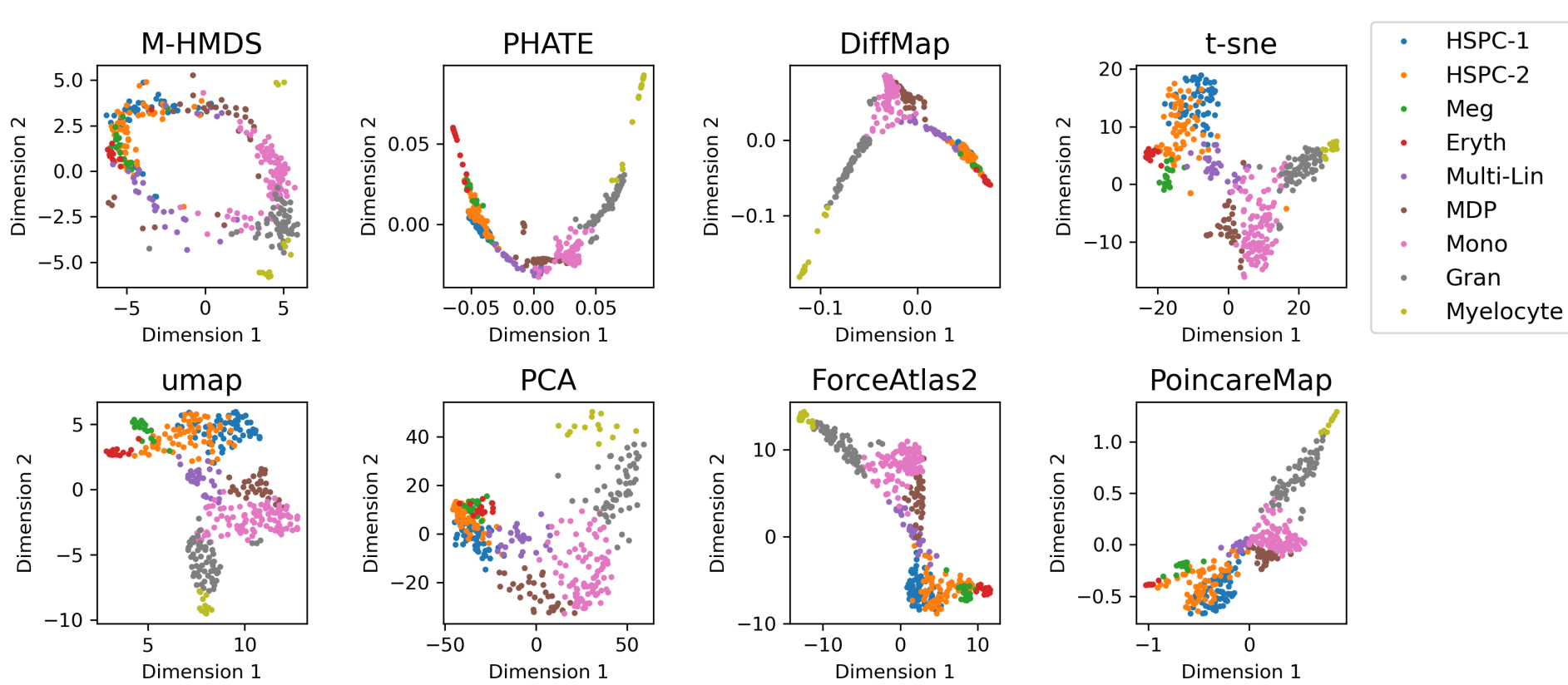

Figure S16: Embedding of the mouse myelopoesis dataset (Olsson), in 2-d space.

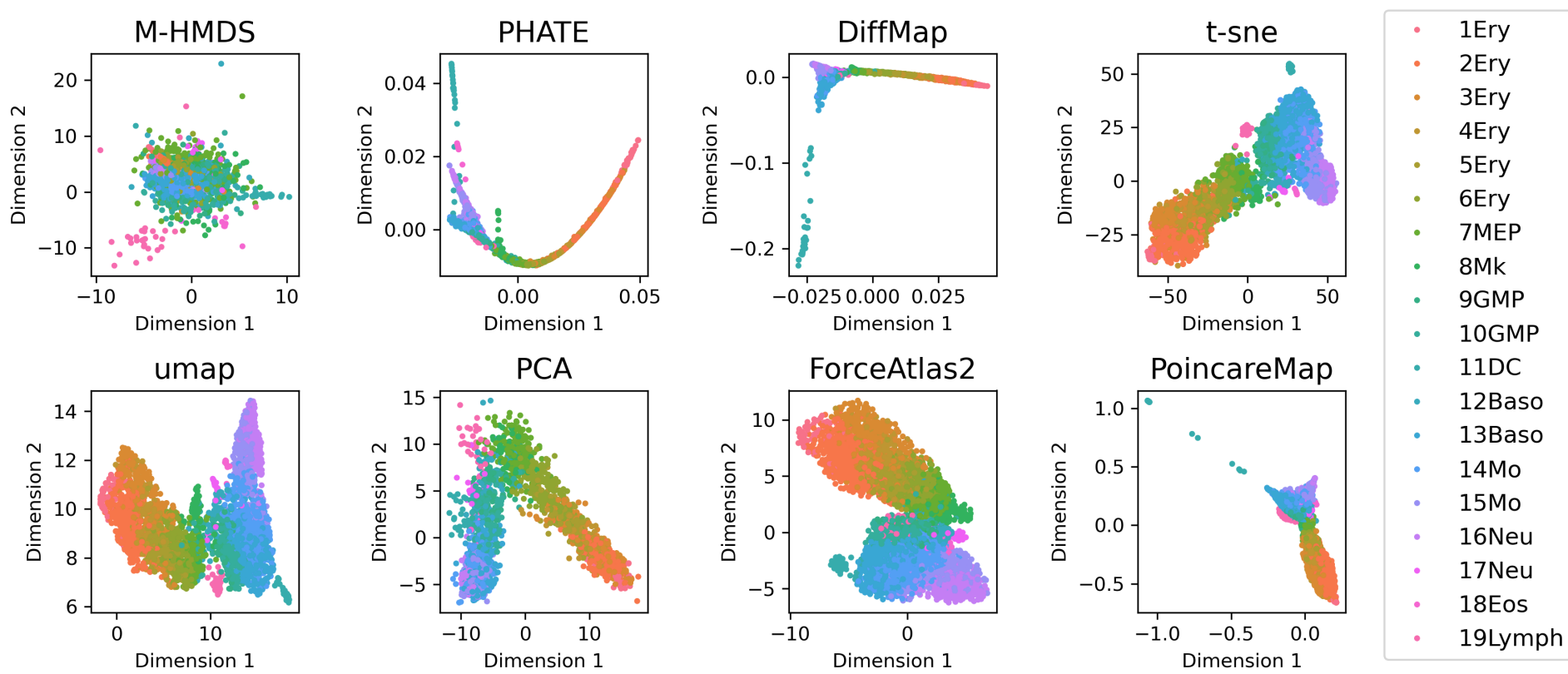

Figure S17: Embedding of the mouse myeloid progenitors dataset (Paul), in 2-d space.

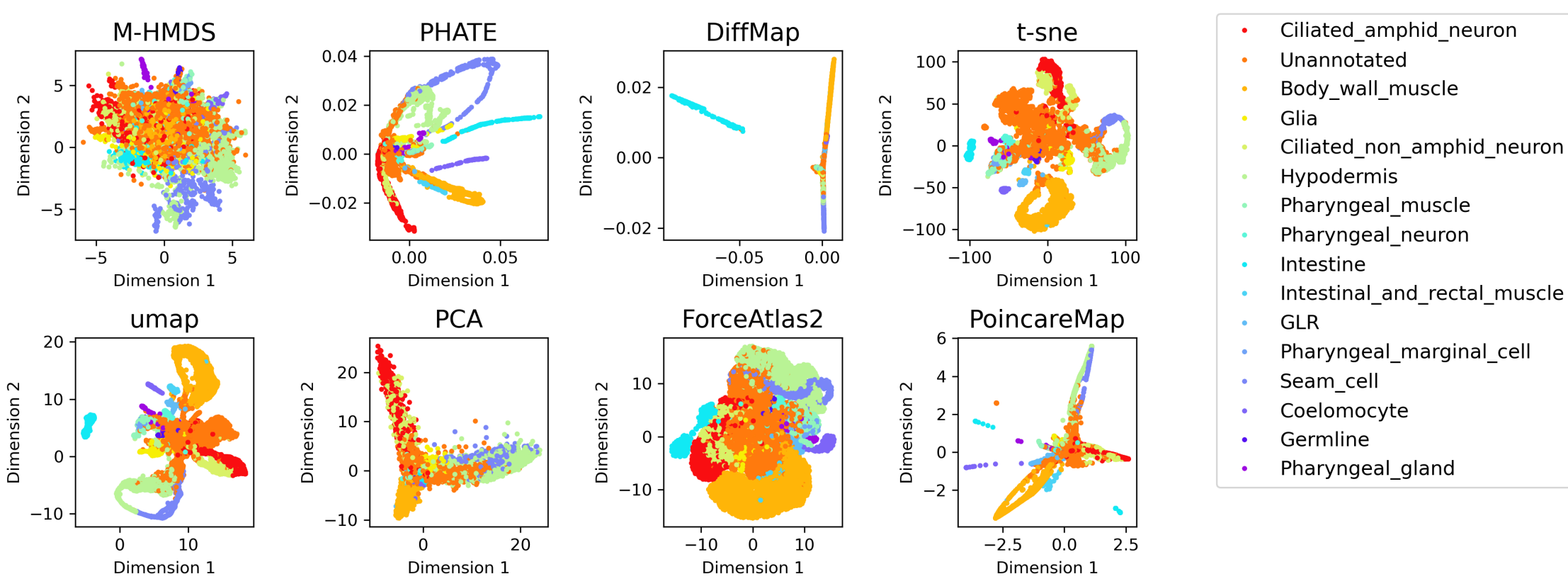

Figure S18: Embedding of the 10,000 sample subset of *C. elegans* dataset, in 2-d space, colored by cell types.

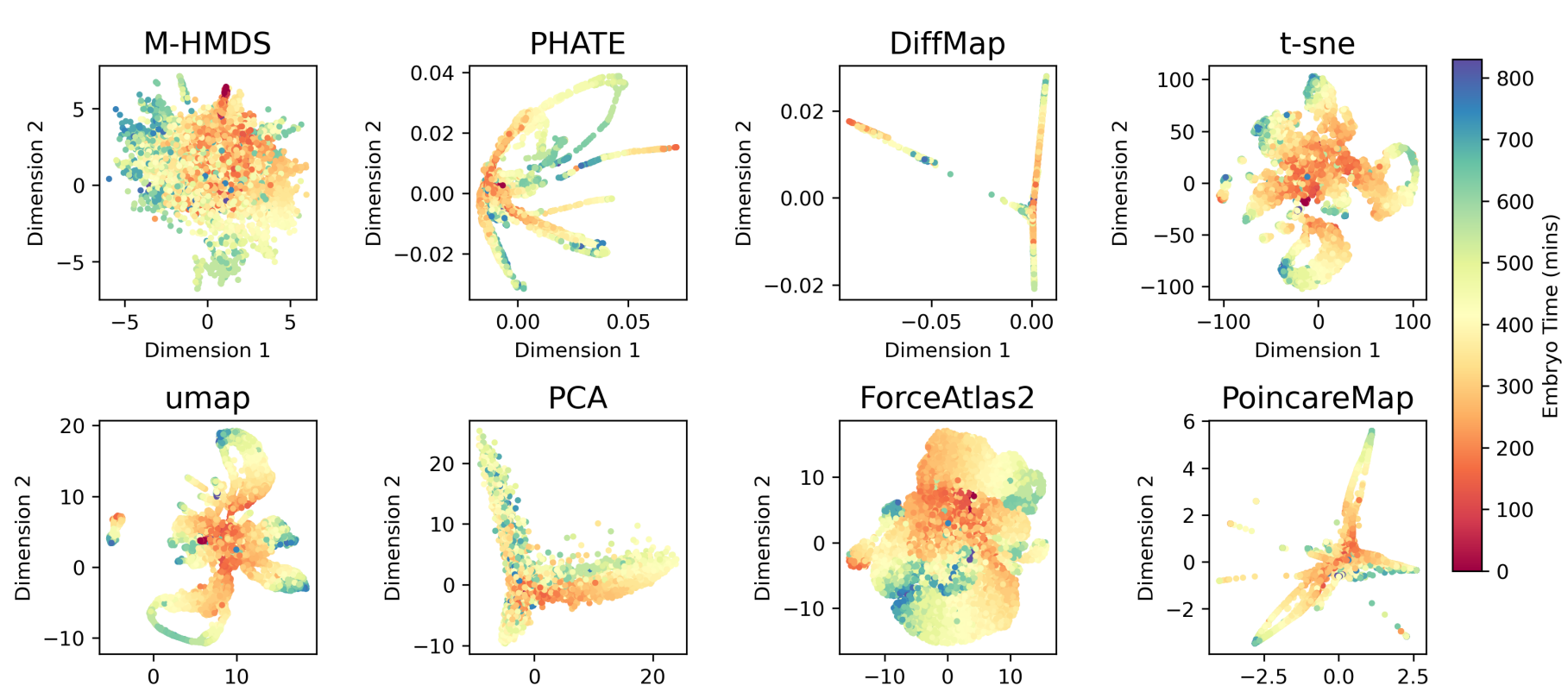

Figure S19: Same as Fig. (S18), but colored by embryo time.

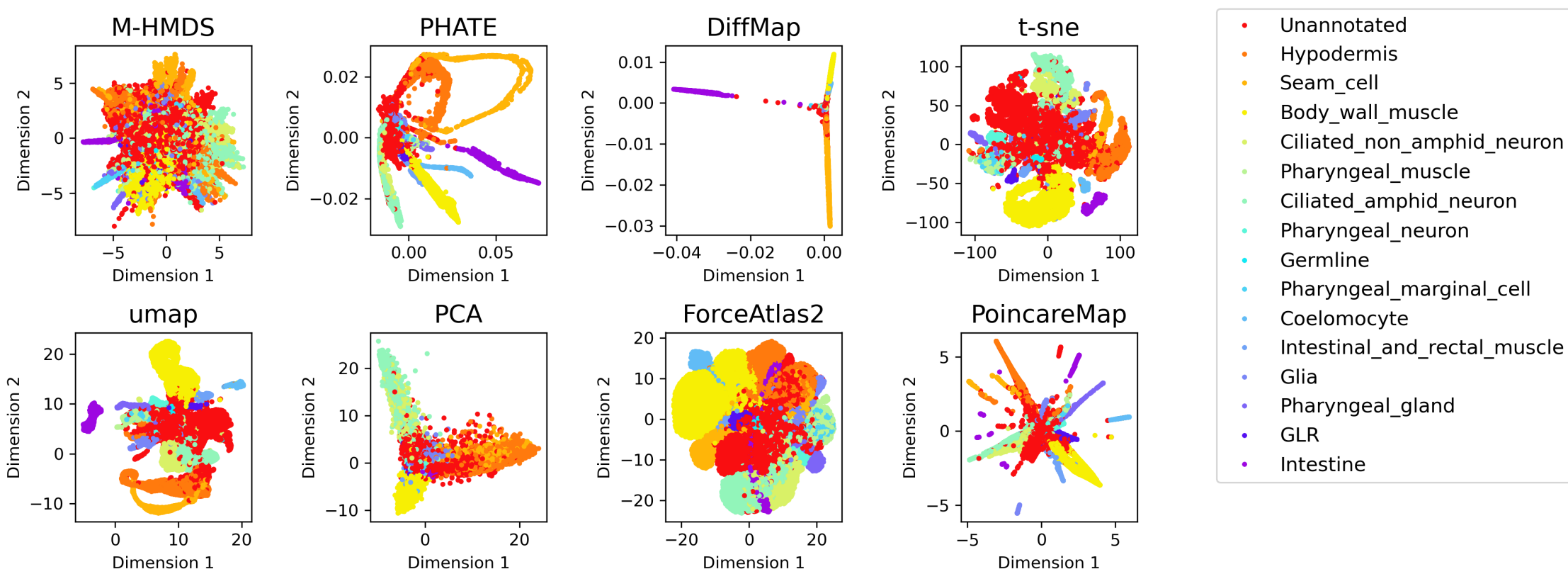

Figure S20: Embedding of the 40,000 sample subset of *C. elegans* dataset, in 2-d space, colored by cell types.

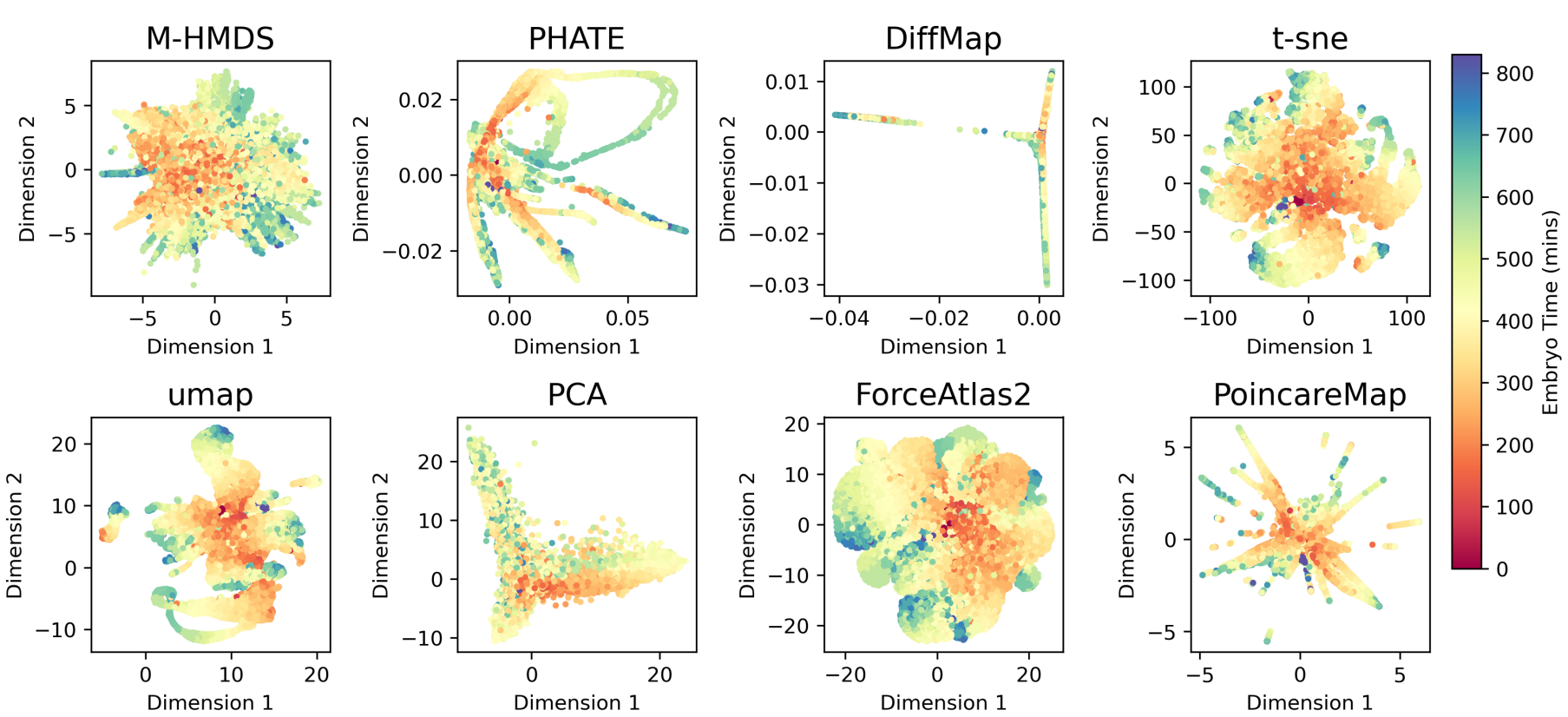

Figure S21: Same as Fig. (S20), but colored by embryo time.
